# Dr. Julius Pohlman

**Published:** 1911-01

**Authors:** 


### OBITUARY

Dr. Julius Pohlman, of Buffalo, died at his home at No. 404 Franklin Street, December 6, 1910, after a short illness, aged 62 years.

Dr. Pohlman was born in Hamburg, Germany, January 26, 1848. In 1872 he came to Buffalo and later entered the Medical Department of the University, where he was graduated in the class of 1883. After his graduation he was appointed Professor in Physiology, which position he held until 1898. He then entered upon the practice of ophthalmology, which he continued until his last illness. He married Lily Bulloch of Glasgow, Scotland, June 27, 1898.

Dr. Pohlman was a member of the American Academy of Political and Social Science, fellow member of the Geological Society, National Geographical Society and a number of other social and scientific societies. He was also a member of the Buffalo Society of Natural Sciences from 1882 to 1890.

Dr. Pohlman is survived by his wife and five children by a previous marriage, Mrs. George Love, Mrs. D. E. Smith, Jenny Pohlman, Julius, Jr., and Dr. August Pohlman, of Bloomington, Indiana.

